# Genome-Wide Identification and Characterization of Tyrosine Kinases in the Silkworm, *Bombyx mori*

**DOI:** 10.3390/ijms19040934

**Published:** 2018-03-21

**Authors:** Songzhen He, Xiaoling Tong, Minjin Han, Yanmin Bai, Fangyin Dai

**Affiliations:** State Key Laboratory of Silkworm Genome Biology, Key Laboratory of Sericultural Biology and Genetic Breeding, Ministry of Agriculture, Southwest University, Chongqing 400715, China; szhe@swu.edu.cn (S.H.); xltong@swu.edu.cn (X.T.); minjinhan@126.com (M.H.); baibai666@email.swu.edu.cn (Y.B.)

**Keywords:** silkworm, tyrosine kinase, characterization, metazoans, expression pattern

## Abstract

The tyrosine kinases (TKs) are important parts of metazoan signaling pathways and play significant roles in cell growth, development, apoptosis and disease. Genome-wide characterization of TKs has been conducted in many metazoans, however, systematic information about this family in Lepidoptera is still lacking. We retrieved 33 TK-encoding genes in silkworm and classified them into 25 subfamilies by sequence analysis, without members in AXL, FRK, PDGFR, STYK1 and TIE subfamilies. Although domain sequences in each subfamily are conserved, TKs in vertebrates tend to be remarkably conserved and stable. Our results of phylogenetic analysis supported the previous conclusion for the second major expansion of TK family. Gene-Ontology (GO) analysis revealed that a higher proportion of *Bm*TKs played roles in binding, catalysis, signal transduction, metabolism, biological regulation and response to stimulus, compared to all silkworm genes annotated in GO. Moreover, the expression profile analysis of *BmTKs* among multiple tissues and developmental stages demonstrated that many genes exhibited stage-specific and/or sex-related expression during embryogenesis, molting and metamorphosis, and that 8 *BmTKs* presented tissue-specific high expression. Our study provides systematic description of silkworm tyrosine kinases, and may also provide further insights into metazoan TKs and assist future studies addressing their functions.

## 1. Introduction

Tyrosine kinases (TKs) are a large and diverse protein superfamily containing canonical catalytic domains. This superfamily is related to serine/threonine kinases, and is capable of regulating multiple cellular pathways [1]. TKs were first identified 39 years ago, and were found to catalyze tyrosine phosphorylation by transferring the γ-phosphate of ATP (adenosine triphosphate) to tyrosine residues on protein substrates [2,3,4,5,6]. According to the presence or absence of trans-membrane domains, TKs are categorized into two major classes, i.e., RTKs (receptor TKs) and CTKs (non-receptor TKs or cytoplasmic TKs). In general, RTKs phosphorylate endo-cellular target proteins in respond to extra-cellular ligands to activate the signal transduction cascades, while CTKs are likely to transmit the intracellular pTyr (phosphotyrosine) signals [7,8]. In addition to the highly conserved TK domains, TK proteins also contain various functional protein domains interacting with other components within signal transduction pathways [9]. Based on kinase domain sequence and protein secondary structure, TKs are generally grouped into 30 subfamilies [10,11].

Compared to many serine/threonine kinases capable of regulating processes of both multi-cellular and unicellular organisms, the functions of TKs primarily involve the regulation of multi-cellular organisms [1,10,12]. TKs mediate the signal transduction events of one of the few signal transduction pathways that are conserved throughout the metazoans [13]. Tyrosine phosphorylation plays vital and ubiquitous roles in multi-cellular biology processes, including tissue differentiation, growth development, immune responses, cell adhesion, motility and apoptosis [9,14,15,16,17,18,19]. Therefore, mutation and malfunction of tyrosine kinase genes result in many diseases such as cancer and diabetes [20,21,22,23]. In history, TKs defined the prototypical class of oncogenes involved in numerous human malignancies [10]. Now, TKs have been considered to be well-verified targets for cancer therapy [24,25]. TK genes are also implicated in congenital disorders such as dwarfism and hereditary lymphedema [15,26,27,28]. At present, TKs have been the subject of a growing number of studies in physiology and pathology, including inflammation, autoimmunity, neuro-degeneration and infectious diseases [24,25].

Physiological effects and in vivo regulatory mechanisms of TKs have been interesting topics of research in recent years [29,30]. After years of research, TKs have been characterized in masses of species, and genome-wide systematic identification and characterization of this protein superfamily has also been conducted in many metazoans, including humans, amphioxus, zebra-fish and green anole lizard [10,11,31,32,33], providing numerous important insights into the structure, function, and regulation of TKs. Some important progress has also been made in the study of silkworm TKs recently [34,35]. However, the systematic information on TK protein superfamily in the Lepidoptera is still lacking.

The silkworm (*Bombyx mori*) is an economically important lepidopteran insect, and has also been considered as a model organism for physiology, microbiology, genetics and functional genomics analysis [36]. In this study, we systematically identified and characterized the silkworm TK genes, performed phylogenetic analyses in metazoans, and analyzed their spatial/temporal expression patterns. This article provides the first comprehensive resource of silkworm TKs. The systematic identification, naming, orthology and expression analysis of TKs encoded in the silkworm genome would facilitate an understanding of the physiological and developmental function of silkworm TKs. Our findings may also provide insights into metazoan TK genes and would aid future studies on their functions.

## 2. Results and Discussion

### 2.1. Identification of Silkworm TK Proteins

To identify TK proteins in silkworm, the HMMER3.0 package (Available online: http://hmmer.org/) [37] was applied to search in the silkworm protein dataset using the hidden Markov model profile for the TK domain (PF07714). Redundant sequences were manually removed. The presence of TK domains was verified by SMART (Simple Modular Architecture Research Tool, Available online: http://smart.embl-heidelberg.de/) and CDD (Conserved Domain Database, Available online: http://www.ncbi.nlm.nih.gov/Structure/cdd/wrpsb.cgi) search-based online domain analysis. As a result, a total of 33 non-redundant silkworm TK proteins (*Bm*TKs) were retrieved, among that 19 are classified as RTKs and 14 are grouped as CTKs.

Generally, TKs are categorized into 30 subfamilies [10,11,33]. The multiformity of TKs is likely to imply the complicacy of the pTyr-based signaling system [38]. Based on sequence similarity and protein secondary structure, we clustered *Bm*TKs into 25 subfamilies (Table 1), while no members fell into AXL, FRK, PDGFR, STYK1 and TIE subfamilies, which are almost absent in lower metazoans [11]. A total of 6 in these 25 subfamilies have multiple members (2 members in ACK, EPH, RYK and SRC1 subfamilies, 3 members in FGFR and INSR subfamilies, respectively).

In silico analysis showed that the lengths of *Bm*TKs ranged from 184 residues to 3012 residues. The molecular weights ranged from 20.47 kDa to 332.25 kDa. Isoelectric points varied from 4.41 to 8.69 (Table 1). We also investigated gene structures and found that the intron sequences within the ORF (open reading frame) of the *BmTK* genes varied greatly and that the shortest *BmTK* gene (*BmMET*) was only ~2.2 kb (kilo-base), while the longest one was *BmFGFR1* with a ~106.3 kb genomic sequence (Table S1). The other detailed information of these genes, including gene and protein ID, sequences, chromosome location and corresponding human gene name and protein accession No., is summarized in Table S1.

### 2.2. Chromosomal Distribution of the BmTK Gene Family

**Figure 1:**
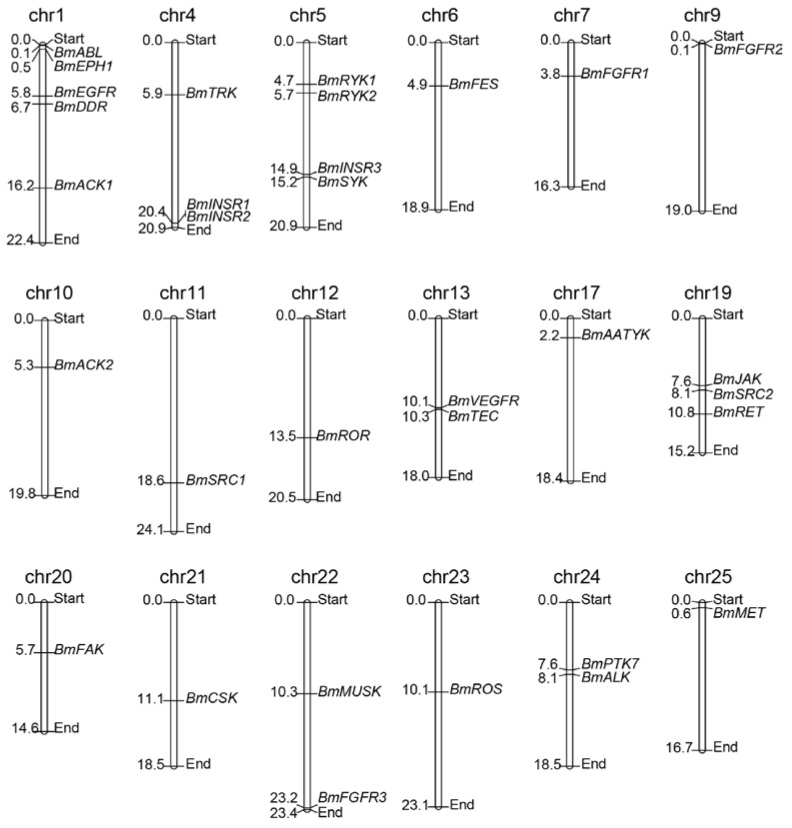
*BmTKs* distribution map on silkworm chromosomes. 32 of the identified silkworm TK genes (i.e., all except *BmEPH2*) were plotted onto chromosomes based on their physical coordinates. The chr indicates chromosome. Chromosomal distances are given in Mb (Mega base).

To determine the distribution and contexts of the *BmTK* genes on silkworm chromosomes, the TK genes were mapped on chromosomes according to their chromosomal coordinates from the silkworm genome database (Available online: http://sgp.dna.affrc.go.jp/KAIKObase/). Chromosomal distribution analysis showed that 32 *BmTKs* are assigned to 18 of the 28 silkworm chromosomes (Figure 1, Table 1 and Table S1), while the other one (*BmEPH2*) does not have location information on chromosomes. The distribution of *BmTK* genes is widespread and scattered on the chromosomes. 11 chromosomes respectively have only one *BmTK* gene, chromosome 1 has the largest number of *BmTK* genes (5 genes), followed by chromosomes 5 (4 genes), chromosomes 4 and 19 respectively have 3 *BmTK* genes, and 2 *BmTK* genes are on chromosomes 13, 22 and 24, respectively (Figure 1). No TK genes closely arrange in tandem (without other genes interrupted between them) on chromosomes, only *BmINSR1* and *BmINSR2* located tandemly on chromosomes but with one other genes between them, that is different from the cases in higher animals which have been systematically studied [10,11,33]. Genome duplication events are thought to contribute in the expansion of gene families [39,40]. It was reported that a large number of TK genes of higher metazoans likely originated from whole genome duplication events [10,11,19,32,33], and many TK genes arranging in tandem may be retained following these events. In contrast, there is no evidence of whole genome duplication events in the silkworm, and given the much less number of TK genes compared with the higher metazoans, it is not hard to understand the absent of the case of closely tandem arrangement of *BmTK* genes.

### 2.3. Comparative Analysis of TKs within Metazoans

To investigate and evaluate the orthologous relationships of TKs, orthology analysis was performed, and a list of orthologous genes between silkworm and humans as well as the protein sequence identities of kinase domains is shown in Table 2. Despite the vast difference between silkworm and humans, the average of sequence identity between their orthologous TK domains is 52%, and the highest sequence identity is actually as high as 77% (ABL) while the lowest sequence identity is 29% (AATYK), suggesting that the TK domain sequences are generally highly conserved and that the degree of variation among TK domain sequences of different subfamilies varies greatly.

Like many other multi-gene families, the TK family shows differential expansion and it was proposed that there are two major expansions in this gene family. The first expansion seems to have occurred before the segregation between the poriferans and the other metazoans, the second occurred around the split between the cyclostomes and the gnathostomes [41,42,43]. It was found that different TK subfamilies differ significantly in sequence conservation (Table 2). 

**Figure 2:**
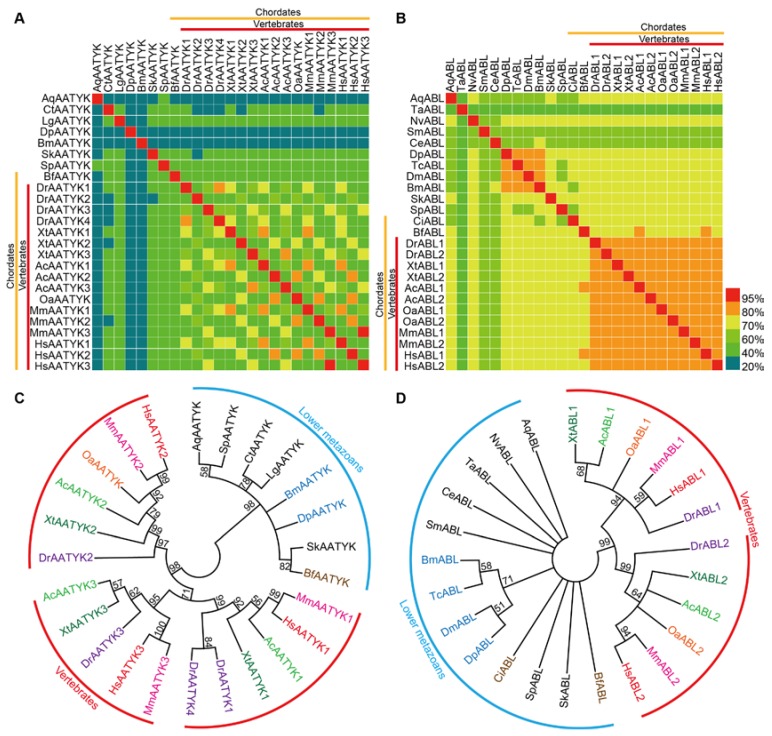
Sequence identity matrices and phylogenetic analysis of AATYK and ABL subfamilies members in metazoans. (**A**,**B**) Sequence identity matrices. The yellow and red lines on the X and Y axes indicate chordates and vertebrates, respectively. (**C**,**D**) TK domain sequence-based phylogenetic trees. The ML (Maximum-Likelihood) trees are based on multiple sequence alignments of TK domains from AATYK and ABL subfamilies. Bootstrap values below 50 are not shown. Chordates and arthropods are indicated by different colors. The red arcs indicate the different protein classifications of the higher chordates (i.e., vertebrates). The blue arcs indicate the proteins of lower metazoans. Corresponding species names for the abbreviations are listed in Table S2.

To study phylogenetic relationships of TK subfamilies with different sequence conservation, we performed sequence identity analysis and phylogenetic analysis for ABLs and AATYKs (subfamilies with the highest and the lowest sequence identities in our analysis above, respectively) in silkworm and 20 other representative metazoan species (Table S2), respectively. Based on sequence identity values and sequences of TK domains, matrices and ML (maximum likelihood) trees were constructed (Figure 2).

Similar to the identity values between silkworm and humans, among all species used for analysis (some species do not have the ABL or AATYK), the sequence identity values of TK-domains in AATYK are relatively low (mean ~51.6%), while the sequence identity values of ABL TK-domains are quite high (mean ~75.2%). Higher sequence identity values are present among the higher chordates (i.e., vertebrates) (Figure 2A,B), indicating that TKs in vertebrates tend to be remarkably conserved and stable. In each of the ML trees, the higher metazoans were clustered into a major clade, and divided into sub-clades for different gene copies of the subfamily, while the lower animals clustered together (Figure 2C,D). Interestingly, two chordates, i.e., *Bf* (*Branchiostoma floridae*; Cyclostome, Cephalochordata, Leptocardii) and *Ci* (*Ciona intestinalis*; Cyclostome, Urochordata, Ascidiacea), have the same gene copy numbers as lower animals, and were clustered with lower animals rather than with other higher chordates (vertebrates). This is similar to the case in the matrices that there are almost no high identity values between *Bf*/*Ci* and vertebrates (Figure 2A,B). These results reveal that the TK domains (at least in AATYKs and ABLs) of invertebrate chordates are more closely similar to lower animals, and suggest that there are expansion and sequence variation events in TK family of vertebrates after segregating from the other metazoans, which are around the period of split between the cyclostomes and the gnathostomes. This supports the previous conclusion [41,42] of the time for the second major expansion of the TK family.

### 2.4. Gene Ontology Analysis of the BmTKs

**Figure 3:**
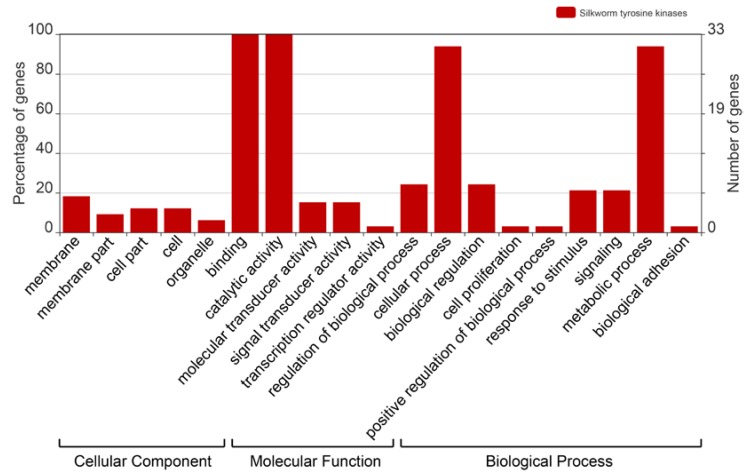
Gene Ontology (GO) categories of *BmTK* genes. This analysis was visualized with an online tool, WEGO (Available online: http://wego.genomics.org.cn/).

To survey the functions of the *Bm*TKs, GO (Gene Ontology) annotation was obtained from two silkworm genome databases (Available online: http://sgp.dna.affrc.go.jp/KAIKObase/ and Available online: http://www.silkdb.org) to construct GO graphs. As shown in the GO graph, all *Bm*TKs (33, 100%) are involved in catalytic activity and binding (Figure 3, Table S3), that are consistent with the primary protein functions. The results also showed that the *Bm*TKs were involved in diverse biological processes and predominantly participated in metabolic process and cellular process (31, ~93.9%) (Figure 3, Table S3). Compared to a wide range of genes with GO annotations, a higher proportion of *BmTK* genes played roles in catalytic activity, binding, signal transducer activity, molecular transducer activity, metabolic process, biological regulation, response to stimulus and so on (Figure S1).

### 2.5. Spatial and Temporal Expression Profile of the BmTK Genes

**Figure 4:**
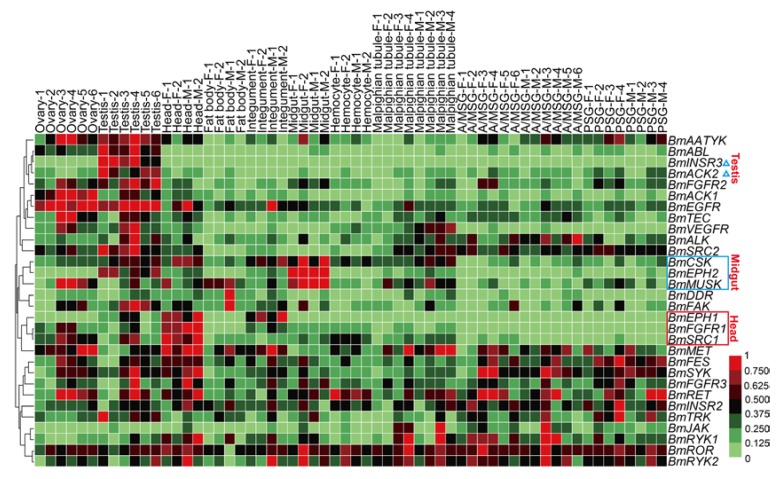
Microarray-based gene expression profiling of *BmTK* genes in multiple tissues. Biological repeats of each tissue are indicated with different numerals. F: female, M: male, A/MSG: anterior/median silk gland, PSG: posterior silk gland. Blue triangles indicate the genes with testis-specific expression. Red box indicates the genes with higher expression in the head. Blue box indicates the genes with high expression in the mid-gut.

Compared to the static characteristics including genomic locations, protein structures, sequence similarities and phylogenetic features, the gene expression patterns provide a more vivid image of the functions and biological activities of genes. To view the expression profile of *BmTK* genes across various tissues, we used the microarray gene expression dataset from SilkDB (Available online: http://www.silkdb.org), which contains normalized gene expression levels across 10 tissues. A total of 30 genes were detected by the tissue microarray (Figure 4, Table S4). Almost half of the genes are highly expressed in the gonads of the silkworm. Approximately one-third of the genes are expressed in the silk glands. *BmINSR3* and *BmACK2* exhibit testis-specific expression (Figure 4, blue triangles). 3 *BmTK* genes (*BmSRC1*, *BmFGFR1* and *BmEPH1*) show higher expression in the head (Figure 4, red box), *BmEPH1* expression is also present in the integument, and *BmSRC1* is also expressed in the gonads. 3 genes (*BmMUSK*, *BmEPH2* and *BmCSK*) are mainly expressed in the mid-gut (Figure 4, blue box). The diverse expression profiles indicate that some *Bm*TK proteins are involved in specific physiological functions in specific tissues.

**Figure 5:**
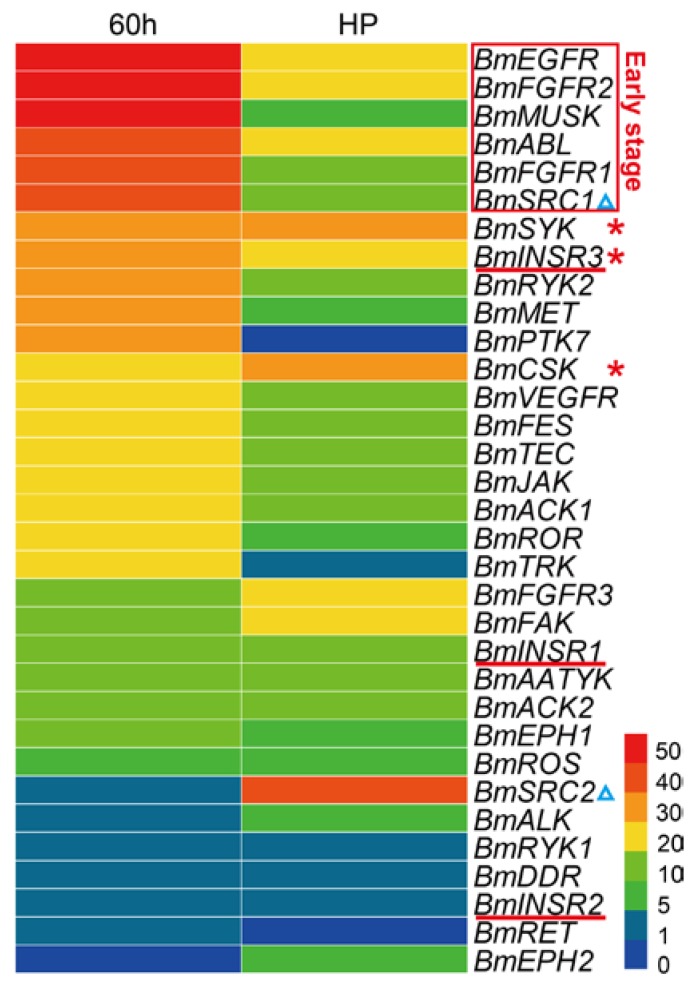
Gene expression profiling of *BmTK* genes in the embryonic period. RPKM (reads per kilo-base per million mapped reads) of digital gene expression data was used to quantify gene expression. 60 h: embryos hatching for ~60 h (early embryos), HP: embryos at head pigmentation stage (late embryos). Red box indicates the genes with high expression in the early embryos. Red asterisks indicate the genes with relatively high expression in both the early and late embryos. Blue triangles indicate the genes in the *SRC* subfamily. Red lines indicate the genes in the *INSR* subfamily.

Due to the significant cellular functions, previous studies have shown essential roles of the TK families in multiple aspects of embryonic development, including a wide range from early events in fertilization and gastrulation to late events in histogenesis and organogenesis [33]. To view the expression patterns of the *BmTK* genes in the silkworm embryonic period, we used the RNA-seq-based DGE (digital gene expression) dataset of silkworm early embryos (hatching for ~60 h, thoracic and abdominal appendages are just formed) and late embryos (head pigmentation stage, organogenesis has basically been completed). The analysis shows that the majority of genes are highly expressed in the early embryos, while *BmTKs* are generally lowly expressed in the late stage of embryos (Figure 5, Table S5), indicating that the roles of these genes in silkworm early embryos in are more pronounced. More detailedly, the expression of *BmEGFR*, *BmMUSK*, *BmFGFR2*, *BmFGFR1*, *BmABL* and *BmSRC1* is high in the early embryos, but is reduced in late embryos (Figure 5, red box). *BmSYK, BmCSK* and *BmINSR3* are with relatively high expression in both the early and late embryos (Figure 5, red asterisks). Interestingly, although belonging to the same subfamily, *BmSRC1* is highly expressed in the early stage, while *BmSRC2* is highly expressed in the late stage (Figure 5, blue triangles). Similarly, compared to the relatively high expression of *BmINSR3* in both early and late stages, *BmINSR1* and *BmINSR2* show low expressions at both the stages (Figure 5, red lines). These results reveal that *BmTK* genes of the same TK subfamily have diverse functions, or play roles at different stages of silkworm embryonic development. To get a more accurate and complete panorama of gene expression and a better understanding of their roles in silkworm embryonic development, we need to select more intensive time points for investigation in the future research and to continue the follow-up functional study.

**Figure 6:**
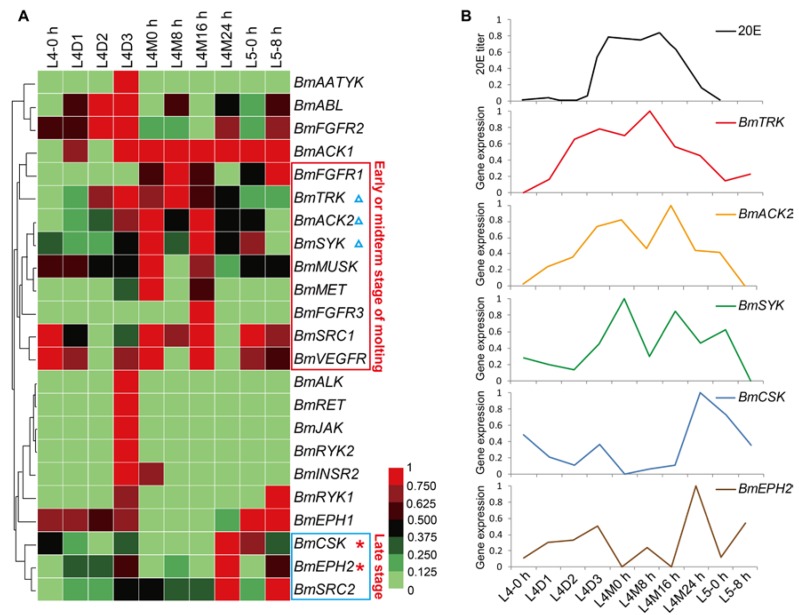
Microarray-based gene expression profiling of *BmTK* genes during the fourth larval molting. 23 *BmTK* genes were detected to be expressed during silkworm larval molting. L: larval (L4-0 h: beginning of the 4th larval instar, L4D1: day 1 of the 4th larval instar), M: molting (L4M0 h: beginning of molting, L4M8 h: 8 h after L4M0 h). (**A**) Red box indicates the genes with high expression at the early or midterm stage of molting. Blue box indicates the genes with high expression at late stage of molting. (**B**) The 20E titer in silkworm haemolymph (from Kiguchi et al. [46]) is shown at the top. *BmTRK*, *BmACK2* and *BmSYK* are with the expression pattern resembling the pulse of 20E (**A**, blue triangles). *BmCSK* and *BmEPH2* might respond to low concentrations of 20E (**A**, red asterisks).

In general, silkworm larvae experience four times of molting and form five larval instars. Larval molts are key physiological processes in silkworm growth. To view the temporal expression profiles of the *BmTK* genes during larval molting, we used the microarray gene expression data around the fourth larval molting, which contains normalized gene expression values across 10 time points from the 4th larval instar to the 5th larval instar. A total of 23 *BmTK* genes were found to be expressed during this period (Table S6), and 13 *BmTK* genes were detected to be highly expressed during larval molting (Figure 6A, from L4M0 h to L4M24 h). *BmACK1* shows high expression across the 7 time points around larval molting, and 9 genes exhibit high expression at the early or midterm stages of molting (Figure 6A, red box), while other 3 genes present high expression at late stage of molting (Figure 6A, blue box). The 20-hydroxyecdysone (20E) is the major regulatory hormone in insects and other arthropods, and mediates developmental transitions such as larval molting and metamorphosis [44,45]. The expression pattern of *BmTRK*, *BmACK2* and *BmSYK* resembles the changing titer of 20E in silkworm haemolymph (Figure 6B), and the expression of *BmCSK* and *BmEPH2* appears to respond to low concentrations of 20E (Figure 6B), suggesting that the expression of these genes might be regulated by 20E.

**Figure 7:**
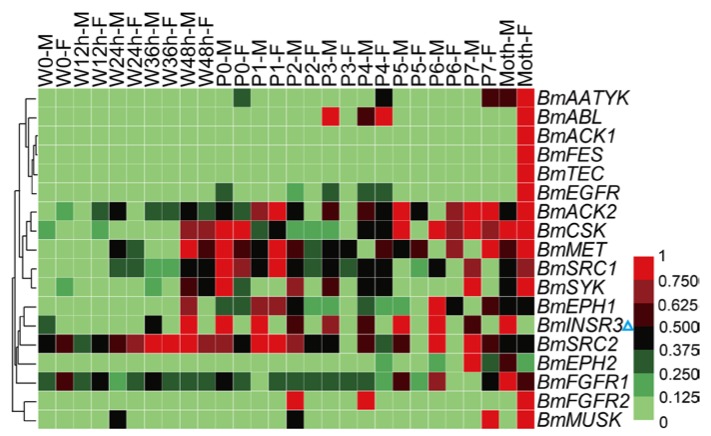
Microarray-based gene expression profiling of *BmTK* genes during silkworm metamorphosis. 18 *BmTK* genes were detected to be expressed during silkworm metamorphosis. W: Wandering (W0: beginning of wandering for spinning, W12 h: 12 h after W0), P: pupation (P0: beginning of pupation, P1: one day after P0), M: male, F: female. Blue triangle indicates the gene with male-specific expression.

The silkworm is an economically-important insect for silk production. Metamorphosis is the most crucial physiological stage of silkworm. Before this stage, silkworms spin cocoons to protect pupae from injury and this behavior also relates to economic benefit. Tissue dissociation and reconstruction occur during the metamorphosis stage, and the physiological status of pupae determines the success rate and synchronization of adult emergence, that are related to the quality and quantity of eggs. To investigate the expression profiles of the *BmTK* genes during metamorphosis, we used the microarray gene expression dataset from spinning to adult stages, which contains normalized gene expression values across 14 time points. A total of 18 *BmTK* genes were found to be expressed during this period (Table S7). Most genes exhibit high expression in the pupal and adult stages (Figure 7), indicating that these genes are involved in silkworm metamorphosis. The hierarchical clustering graph shows that the expression pattern of *BmINSR3* displays sexual dimorphism, which is specifically expressed in male individuals (Figure 7, blue triangle). We also found that *BmINSR3* were expressed specifically in the testis (Figure 4, blue triangle), indicating that this gene may be required for spermatogenesis or testicular development.

Our results have also been supported by studies on the function of orthologous genes in other species. We found that the expression of *BmSRC1*, *BmMUSK* and *BmEGFR* was very high in the early embryos of silkworm (Figure 5, red box). Previous studies in zebra-fish have shown that: the non-receptor tyrosine kinase SRC participated in egg activation during fertilization [47], MUSK played roles in neuromuscular synapse formation [48], and the EGFR receptor tyrosine kinase family affected the development of neural crest in the early stage of embryos [49]. The EPHR family is crucial for retinotectal system pattern, mesenchymal-to-epithelial transition and extracellular matrix assembly [50,51,52,53], and our results showed *BmEPH1* exhibited relatively high expression in the head (Figure 4, red box). The MET receptor tyrosine kinase (ortholog of *Bm*MET, whose encoding gene is highly expressed in the head and fat-body of silkworm, and fat-body is an insect organ functionally homologous to liver) was proved to regulate the development of liver and cerebellum [54,55,56].

## 3. Materials and Methods

### 3.1. Genome-Wide Identification of TK Proteins

Using the hidden Markov model (HMM) of tyrosine kinase domains (TK or Pkinase_Tyr, PF07714), hmmsearch program (HMMER3.0, Available online: http://hmmer.org/ [37], *E*-value ≤ 1 × 10^−1^, score ≥ 0) was applied to identify the putative TK proteins in the silkworm protein dataset downloaded from two silkworm genome databases, KAIKObase (Available online: http://sgp.dna.affrc.go.jp/KAIKObase/) and SilkDB (Available online: http://www.silkdb.org). Redundant sequences were deleted manually. Two online tools, SMART (simple modular architecture research tool, Available online: http://smart.embl-heidelberg.de/) [57] and CDD (conserved domain database, Available online: http://www.ncbi.nlm.nih.gov/Structure/cdd/wrpsb.cgi) [58], were used to verify the TK domains in the predicted proteins. The TK genes were annotated by online BLASTP searches (standard protein BLAST, basic local alignment search tool) against the non-redundant protein database in NCBI (Available online: https://www.ncbi.nlm.nih.gov/).

### 3.2. Chromosomal Distribution and Gene Basic Information

The protein lengths, gene lengths, chromosomal locations, transcription directions and probes ID were obtained from KAIKObase and SilkDB. Isoelectric point values and theoretical molecular weights were calculated online at http://web.expasy.org/protparam/. Based on silkworm chromosomal coordinates of genes from the KAIKObase, the TK genes were mapped on chromosomes according to their order of physical position, and the *BmTKs* distribution map was constructed by MapChart [59].

### 3.3. Sequence Identity Matrix and Phylogenetic Analysis

Tyrosine kinase sequences of humans were retrieved from the previous reports [10]. We carried out pairwise sequence comparison for TK domain sequences of humans and silkworm to reveal sequence identity using BLASTP.

ABLs and AATYKs of other 19 representative metazoans were obtained from the previous reports (amphioxus [32] and zebra-fish [33]) or identified by BLASTP searches (Available online: ftp://ftp.ncbi.nlm.nih.gov/blast/executables/blast+/LATEST/) against protein sequences of the human ABLs and AATYKs. The information of these species is listed in Table S2. The protein sequences were downloaded from NCBI (Available online: ftp://ftp.ncbi.nlm.nih.gov/genomes/) and Ensembl (Available online: http://www.ensembl.org), and were verified using the online program SMART.

Pairwise sequence comparison of TK domain sequences of ABLs and AATYKs in the 21 metazoans was performed using BLASTP to reveal sequence identity. The sequence identity matrices were generated using the heat map illustrator (HemI) software package (version 1.0.3.3, Huazhong University of Science and Technology, Wuhan, China) [60].

Multiple sequence alignments for TK domains of ABLs and AATYKs from the 21 metazoans were performed by the MUSCLE (multiple sequence alignment) program [61,62], and unrooted phylogenetic trees were constructed by the maximum-likelihood method [63,64] with a bootstrap of 1000 replicates.

### 3.4. Gene Ontology Annotation

Gene Ontology (GO) categories of genes were obtained from two silkworm genome databases (Available online: http://sgp.dna.affrc.go.jp/KAIKObase/ and Available online: http://www.silkdb.org), and was visualized with WEGO (web gene ontology annotation plotting, Available online: http://wego.genomics.org.cn/) [65].

### 3.5. Expression Profiling Based on DGE Sequencing and Microarray Data

Based on the microarray gene expression data of 10 tissues on Day 3 of the fifth instar [66], we investigated the spatial expression pattern of *BmTK* genes. Each tissue was analyzed using at least two biological repeats, including head, fat body, midgut, Malpighian tubule, integument, hemocyte, ovary, testis, A/MSG (anterior/median silk gland) and PSG (posterior silk gland). Each biological repeat was a mixture from multiple individuals. As the previously-described protocol [67], four house-keeping genes (encoding proteasome β subunit, eIF 3A subunit 5, eIF-3 subunit 4 and eIF 4A) were used to normalize raw microarray data by a linear normalization method. The normalized values were then processed by the min-max normalization, and were used to generate hierarchical clustering graph based on a Pearson correlation distance metric and average linkage clustering using the HemI software package [60].

To elucidate the expression pattern of *BmTK* genes during silkworm larval molting and metamorphosis, we analyzed the microarray gene expression data at 10 developmental time points around the fourth larval molting and 14 developmental time points around metamorphosis. The raw microarray data were processed as described above. If the normalized signal intensity value of a gene was less than 400, the gene was considered to be unexpressed. Gene expression microarray data on L5D3 (day 3 of the 5th larval stage) were used as a control. The ratio of the signal intensity values of each gene at each developmental time point to that in the L5D3 was used to investigate dynamic changes of the expression level. The ratio values were then processed by the min-max normalization, and were used to generate hierarchical clustering graphs by the HemI software package as described above.

Based on DGE (digital gene expression) sequencing data of embryos hatching for ~60 h (early embryos, thoracic and abdominal appendages are just formed) and embryos at HP (head pigmentation) stage (late embryos, organogenesis has basically been completed), we investigated the expression pattern of *BmTK* genes in the embryonic period. RPKM (reads per kilo-base per million mapped reads) was used to quantify gene expression. The hierarchical clustering graph was generated using the HemI software package.

## 4. Conclusions

Tyrosine kinases (TKs), important components of signaling pathways, play key roles in cell growth, development, apoptosis and disease. We conducted a genome-wide analysis in silkworm and identified 33 TK-encoding genes belonging to 25 subfamilies. Sequence identity matrix analysis suggested that TKs in vertebrates tended to be remarkably conserved and stable. Phylogenetic analysis suggested that expansion and sequence variation events occur in TK family in vertebrates. GO analysis revealed that more *Bm*TKs played roles in binding, catalysis, signal transduction, metabolism, biological regulation and response to stimulus, compared to all silkworm genes annotated in GO. Expression pattern analyses revealed the tissue-specific, stage-specific, or sex-dimorphic expressions of the *BmTK* genes. These findings complement systematic information on TK family of silkworm in Lepidoptera, and increase our understanding of metazoan TKs. The follow-up functional studies are required for a better understanding of the roles of TKs in regulation of key growth and developmental processes.

## Figures and Tables

**Table 1:** Inventory of TK genes in the silkworm genome.

Type	Gene	Protein Length	Molecular Weight	Isoelectric Point	Chromosome
RTKs	*BmDDR*	656	75,616.43	8.24	chr1
	*BmEGFR*	1448	161,311.64	5.82	chr1
	*BmEPH1*	1006	111,746	5.78	chr1
	*BmTRK*	578	64,646.3	5.43	chr4
	*BmRYK1*	597	65,058.48	8.49	chr5
	*BmRYK2*	542	60,476.73	6.98	chr5
	*BmFGFR1*	856	97,292.72	5.86	chr7
	*BmFGFR2*	616	69,437.85	5.61	chr9
	*BmROR*	971	109,667.49	8.05	chr12
	*BmVEGFR*	1366	155,204.04	5.85	chr13
	*BmAATYK*	3012	332,254.39	4.41	chr17
	*BmRET*	807	91,551.54	6.24	chr19
	*BmFGFR3*	910	134,082.47	8.65	chr22
	*BmMUSK*	638	72,945.69	5.88	chr22
	*BmROS*	2464	276,014.61	6.56	chr23
	*BmALK*	1698	187,163.1	6.67	chr24
	*BmPTK7*	184	21,040.15	6.31	chr24
	*BmMET*	739	83,289.74	5.83	chr25
	*BmEPH2*	184	20,469.51	4.85	unknown
CTKs	*BmABL*	1247	136,157.76	8.31	chr1
	*BmACK1*	1136	127,807.11	6.84	chr1
	*BmINSR1*	985	107,739.52	8.53	chr4
	*BmINSR2*	1022	112,004.68	6.67	chr4
	*BmINSR3*	1472	164,583.02	6.4	chr5
	*BmSYK*	803	90,867.3	6.34	chr5
	*BmFES*	867	98,133.68	8.38	chr6
	*BmACK2*	911	102,923.58	6.89	chr10
	*BmSRC1*	521	59,837.59	7.22	chr11
	*BmTEC*	657	74,694.1	8.49	chr13
	*BmJAK*	1074	123,869.62	6.75	chr19
	*BmSRC2*	507	58,373.31	6.21	chr19
	*BmFAK*	1524	169,553.79	7.39	chr20
	*BmCSK*	589	64,243.5	8.31	chr21

RTKs indicate receptor tyrosine kinases; CTKs indicate cytoplasmic (non-receptor) tyrosine kinases.

**Table 2:** Orthology of silkworm and human TKs with names, protein accession numbers, and sequence identity of kinase domains.

Type	Silkworm Gene Name	Protein Accession	Human Gene Name	Protein Accession	Identity
RTKs	*BmAATYK*	XP_021203756.1	*AATYK*	NP_004911	29%
	*BmALK*	XP_012552890.1	*ALK*	NP_004295	61%
	*BmDDR*	NP_001108469.1	*DDR2*	NP_006173	42%
	*BmEGFR*	XP_004929799.2	*ERBB4*	NP_005226	65%
	*BmEPH1*	XP_021207962.1	*EPHA5*	P54756	72%
	*BmEPH2*	Gene016321	*EPHA2*	NP_004422	47%
	*BmFGFR1*	NP_001037558.1	*FGFR3*	NP_000133	64%
	*BmFGFR2*	XP_004922880.2	*FGFR1*	AAA35835	48%
	*BmFGFR3*	BGIBMGA010869	*FGFR1*	AAA35835	37%
	*BmMET*	XP_004925752.1	*RON*	NP_002438	40%
	*BmMUSK*	NP_001186538.1	*MUSK*	NP_005583	62%
	*BmPTK7*	XP_021209072.1	*PTK7*	NP_002812	39%
	*BmRET*	NP_001164049.1	*RET*	P07949	44%
	*BmROR*	XP_021203906.1	*ROR1*	NP_005003	55%
	*BmROS*	XP_012551361.1	*ROS1*	NP_002935	59%
	*BmRYK1*	XP_021207735.1	*RYK*	AAB26341	44%
	*BmRYK2*	XP_004932386.1	*RYK*	AAB26341	50%
	*BmTRK*	XP_004929369.2	*NTRK2*	NP_006171	46%
	*BmVEGFR*	XP_012547297.1	*VEGFR1*	NP_002010	43%
CTKs	*BmABL*	XP_012551486.1	*ABL1*	NP_005148	77%
	*BmACK1*	XP_004933204.2	*ACK1*	NP_005772	47%
	*BmACK2*	XP_004930430.1	*ACK1*	NP_005772	58%
	*BmCSK*	XP_012548803.1	*CSK*	NP_004374	57%
	*BmFAK*	XP_021207025.1	*FAK*	NP_005598	59%
	*BmFES*	XP_012549644.1	*FES*	NP_001996	57%
	*BmINSR1*	XP_021203435.1	*INSR*	NP_000199	44%
	*BmINSR2*	XP_021203450.1	*INSRR*	AAC31759	35%
	*BmINSR3*	NP_001037011.1	*IGF1R*	NP_000866	71%
	*BmJAK*	XP_021206252.1	*JAK2*	NP_004963	34%
	*BmSRC1*	XP_004932912.1	*FYN*	NP_002028	57%
	*BmSRC2*	XP_012549151.1	*FYN*	NP_002028	70%
	*BmSYK*	XP_004930074.1	*ZAP70*	A44266	48%
	*BmTEC*	XP_021204775.1	*TEC*	NP_003206	58%

## Supplementary Materials

Supplementary materials can be found at http://www.mdpi.com/1422-0067/19/4/934/s1.

## Abbreviations

TK	Tyrosine Kinase
*Bm*	*Bombyx mori*
kDa	kilo-Dalton
RTK	Receptor TK
CTK	Cytoplasmic TK (nonreceptor TK)
