# Supplementary material for: Genome-Wide Identification and Characterization of Tyrosine Kinases in the Silkworm, Bombyx mori
Source: Int J Mol Sci. 2018 Mar 21;19(4):934. doi: 10.3390/ijms19040934 (PMC5979338; doi:10.3390/ijms19040934)
Supplement: Supplementary file 1 [file ijms-19-00934-s001.zip › Supplementary Materials.docx]

**Supplementary Materials: Genome-Wide Identification and Characterization of Tyrosine Kinases in the Silkworm, *Bombyx mori***

**
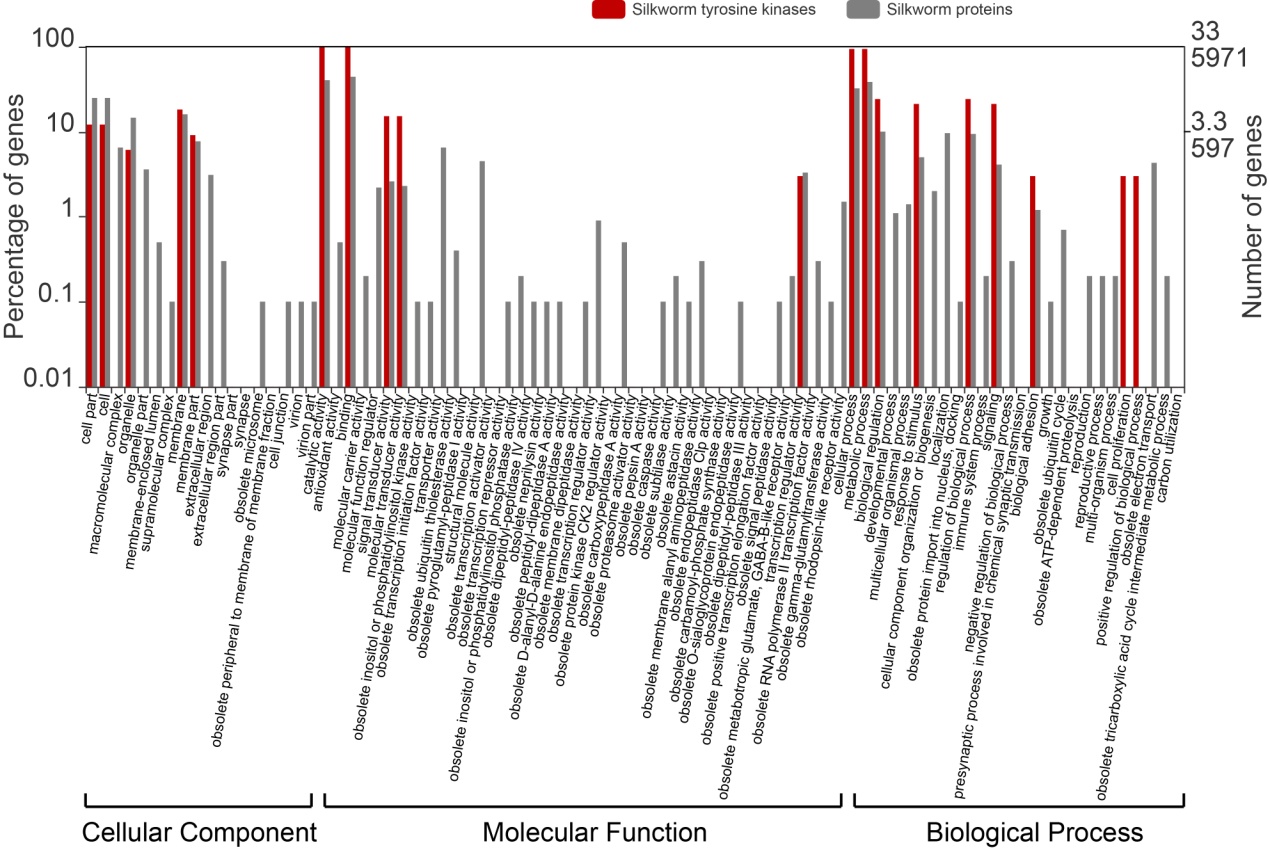
**

**Figure S1.** Gene Ontology categories of *BmTK* genes and genes with GO annotations obtained from SilkDB. This analysis was visualized with WEGO.

**Table S1.** Summary of *BmTK* protein genes. (XLS)

**Table S2.** A list of other metazoan species included in the analysis. (XLS)

**Table S3.** A list for Gene Ontology classification of *BmTK* genes. (XLS)

**Table S4.** Microarray gene expression data of *BmTKs* in multiple tissues of silkworm. (XLS)

**Table S5.** Digital gene expression [data](http://xueshu.baidu.com/s?wd=paperuri%3A%28492a5bd0ba0d1135cdc318d308e4ae77%29&filter=sc_long_sign&sc_ks_para=q%3DAccurate%20Estimation%20of%20Gene%20Expression%20Levels%20from%20DGE%20Sequencing%20Data&sc_us=17936904051661692402&tn=SE_baiduxueshu_c1gjeupa&ie=utf-8) of *BmTKs* in the embryonic period. (XLS)

**Table S6.** Microarray gene expression data of *BmTKs* during the fourth larval molting. (XLS)

**Table S7.** Microarray gene expression data of *BmTKs* during metamorphosis. (XLS)
